# Effects of institutional policies on employees with nonobvious disabilities

**DOI:** 10.4102/ajod.v12i0.1103

**Published:** 2023-03-17

**Authors:** Anthony G. Stacey

**Affiliations:** 1 Wits Business School, Faculty of Commerce Law and Management, University of the Witwatersrand, Johannesburg, South Africa

**Keywords:** ableism, disablism, aversive ableism, discrimination, autoethnography, reasonable accommodation, inclusivity

## Abstract

**Background:**

While legislation protects persons with disabilities against discrimination, decisions taken in line with institutional policies may still have a negative impact on the lived experience of those individuals.

**Objectives:**

The purpose of the study is to evaluate the efficacy of institutional policies, to describe the unintended psychosocial impact of policies and to identify factors that moderate the impact of the policies.

**Method:**

The study adopted an autoethnographic approach involving recollecting life experiences, reading archival and policy documents, reflecting on experiences, articulating lived experiences, deep thought, reviewing and repetition. Activities were carried out as and when appropriate, not necessarily sequentially. The aim was to produce a coherent narrative with credibility, authenticity and integrity.

**Results:**

The results indicate that decisions based on interpretation of policies did not necessarily result in persons with disabilities being fully included in normal academic activities. A disablist institutional culture substantially moderates the intended consequences of institutional policies on the experiences of persons living with disabilities, particularly those that are nonobvious.

**Conclusions:**

Consideration of persons of all abilities should be no different from recognising the diverse needs of persons of different genders, ages, educational backgrounds, financial means, languages and other demographics. A culture of disability prejudice, even among well-meaning individuals, prevents a progressive policy framework from ensuring inclusivity for persons with disabilities.

**Contribution:**

The study demonstrates that a supportive institutional culture is necessary to give effect to disability policies and legislation and to optimise the inclusion of persons with disabilities in the workplace.

## Introduction

The overarching objective of this article is to explore the relationship between employers’ policies and the lived experiences of employees with disabilities, with specific emphasis on nonobvious disabilities. Society at large is alerted to persons’ impairments if their disabilities are evident. Persons with disabilities that are not evident to a casual observer generally receive no consideration for their impairments. Therefore, they are faced with different challenges in society and their workplaces.

This study addresses hermeneutical injustice, when a person’s lived experiences are not well understood by themselves or by others and cannot therefore be adequately articulated (Fricker 2007). My intention is to demonstrate and achieve absolute sincerity, because objectivity would be unrealistic under the circumstances. It will become apparent that I live with acquired disabilities, and the study will consider the effect of workplace policies. I acknowledge that, given the opportunity, my employer would surely present an alternative narrative which does not detract from my lived experience.

The goals of the study are threefold. The first is to evaluate the efficacy of institutional policies. The stated intention of these policies is to guide management decision-making, remove discriminatory barriers and promote access to full participation in all aspects of the institution and facilitate self-representation by persons with disabilities. A second goal is to describe the unintended psychosocial impact of the implementation of policies. Finally, the study seeks to identify factors that moderate the impact of the policies. Persons with disabilities are included as one of the ‘designated groups’ in the country’s *Employment Equity Act* (Republic of South Africa 1998), for whom affirmative action measures must be implemented. The study will not examine the broader policy framework, which includes the White Paper on the Rights of Persons with Disabilities (Department of Social Development 2016) and the Convention on the Rights of Persons with Disabilities (United Nations General Assembly 2006).

My explanations and interpretations may be imperfect because I do not have a medical or legal background. Nevertheless, they are sincere and based on my experiences and understanding. I refer throughout to persons ‘with disabilities’ (in the plural) because multiple interrelated disabilities are not unusual and are my personal experience. When referring to persons with disabilities I include those living with a single disability. I also use the term in preference to ‘disabled persons’ or any other such term because I self-identify as a person with disabilities. I use the term ‘nonobvious’ rather than the narrower term ‘invisible’ so as to include disabilities that may be evident but generally overlooked or unnoticed.

The scope of disability literature includes both *ableism* and *disablism*. These terms are not interchangeable. Bennett (1989) noted that disability rights activists adopted the term ‘ableism’ to refer to an attitude that unduly values those who are able-bodied. However, Wolbring (2008) is critical of a narrow definition of ableism and argues that it is a generic term for a variety of ‘isms’ including racism, sexism, casteism and ageism. Gappmayer (2021) summarises ableism succinctly as ‘the social norm of being able’ (p. 102). In the context of this study, ableism exists when normal is synonymous with ability, while disability is considered abnormal, an aberration, anomalous and requiring restoration to normality. Individual-level discrimination against those with disabilities is referred to as disablism (Bogart & Dunn 2019). Wolbring (2008) suggests that ableism leads to disablism, being discrimination against those that are less able. Disablism is the antithesis of inclusivity and may be more recognisable than ableism. This study is autoethnographic (Ellis, Adams & Bochner 2011) and draws on my experiences of discrimination. Hence, there is a greater focus in this article on disablism.

## Research methods and design

I am employed in a higher education institution in South Africa. The institution is regulated by the *Higher Education Act* (Republic of South Africa 1997), while the statute of the institution defines the mechanisms that give expression to the provisions of the Act. Policies express the institution’s position on a broad range of issues and direct decision-making throughout the institution.

**FIGURE 1:**
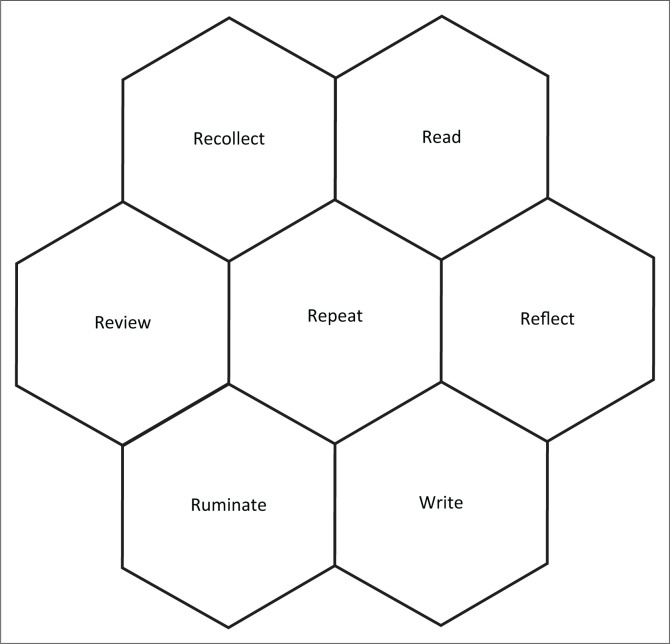
Diagram of the seven ‘R’s of my approach to this study.

Adams, Ellis and Jones (2017) noted that persons who have not experienced cultural and/or institutional oppression cannot articulate the issues in the same ways as those with first-hand experience. They state that autoethnography is the research method that captures what they refer to as ‘insider knowledge’ (p. 3). Researchers now generally accept the method as the amalgamation of autobiography (‘auto’), cultural experiences and artefacts (‘ethno’) plus description and interpretation (‘graphy’) – see, for example, Ellis et al. (2011). The data for this study comprise my lived experiences plus relevant policy documents of my employer. These documents are freely available to internal stakeholders of the institution but are not necessarily easily accessible externally. The autoethnographic approach is not prescriptive or rigidly defined, and the researcher has to adopt specific activities that are appropriate to the subject matter. Figure 1 illustrates the specific activities I undertook in this study.

The principal elements of the method I adopted in this study are as follows:

- **Recollection:** The autoethnographic approach involves documenting specific incidents in my life that have particular significance or have proved transformative in some way. These events are sometimes referred to as ‘epiphanies’ because they can result in profound insights or re-evaluation of the author’s lived experience.
- **Reading:** One can gain richness and insights from reading relevant archival documents and correspondence which comprise important data for the study. The researcher needs to read and familiarise themself with policies and prior research that will be used to analyse the data.
- **Reflection:** The adage that ‘we do not learn from our experiences; we learn by reflecting on our experiences’ captures the role and importance of reflection. This requires self-awareness and consideration of our experiences within a greater context in order to make better sense of them and learn from them:
- **Writing:** The medium of documentation and communication is largely through the written word. There is an onus on the autoethnographer to articulate their lived experiences with such richness and vividness that these resonate with their readers.
- **Rumination:** Analysis of the data requires deep, carefully considered thought and feelings. This does not refer to the psychological meaning of continuously thinking sad or dark thoughts that can prolong or intensify depression. Rumination differs from reflection in that it includes re-experiencing emotional elements of past events.
- **Reviewing:** The purpose of the review is to ensure the appropriate continuity of the narrative and cohesion between consecutive sections of the text to validate judgements. Complementary constructs or interpretations can be identified that may previously have been overlooked.
- **Repetition:** The autoethnographic approach is not a linear sequence of activities. I repeated the various activities as often as necessary, often concurrently, opportunistically and pragmatically, rather than in a predictable and cyclical manner.

I do not identify any of the characters by name out of respect for their confidentiality. However, I make an exception for the late Dr Rob Hawke, to whom I pay tribute for probably saving my life when I was just 13 years old.

## Context

### Childhood head injury

On 29 December 1971, I was attending the 1st Kenilworth Scouts Annual Camp (now known as the Annual Hermanus Camp) at De Mond, Hermanus, Western Cape, South Africa. The daily programme included sailing, canoeing and hiking in the vicinity of the camp, with each camper free to choose which activity to go on under the supervision of one or more adults. That day, I chose to hike up the river gorge in the Zilvermijn Valley, now within the Crystal Kloof Conservancy, some 10 km from the campsite. Dr Rob Hawke, the camp doctor, had a penchant for leading sailing expeditions on the nearby Klein River Lagoon. Fortuitously, on that day he chose to be one of three adult leaders of the Zilvermijn hike with about 10 campers.

While hiking up the gorge and approaching the rock pool, which was to be the lunchtime swimming and picnic stop, we had to climb around a rock face adjacent to a waterfall. The leader of the hike climbed ahead to find a suitable route, while the others in the party waited at the foot of the rock face. The leader accidentally dislodged a large rock, and with only a moment’s notice, I looked up and took a direct blow to the forehead.

**FIGURE 2:**
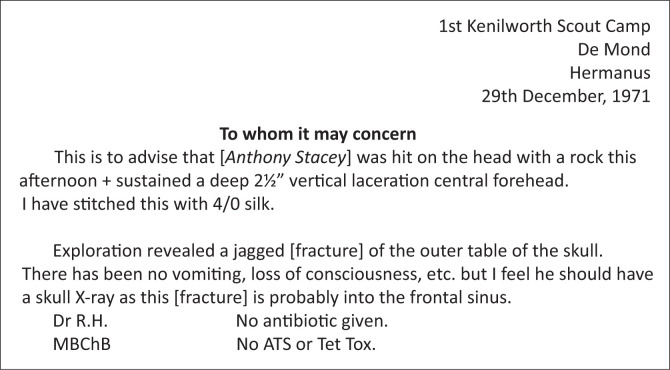
Letter from camp doctor (handwritten).

The camp doctor responded immediately to stop the bleeding by applying direct pressure on the wound. He then cleaned the wound in the river and improvised a bandage by tearing up a beach towel, while the rest of the hiking party had lunch. He returned to the main camp site ahead of the hiking party to make preparations for my return. An older camper, who tragically lost his life years later in the attack on the Westgate shopping mall in Nairobi, carried me down the valley to the main road. Once back in camp, the camp doctor treated me in his tent, before I was driven back home to Cape Town with the referral letter transcribed in Figure 2.

**FIGURE 3:**
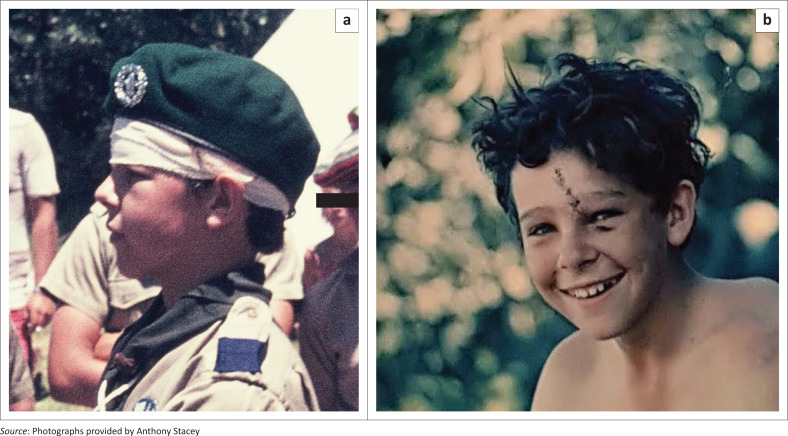
(a) A side view of Anthony Stacey back at camp 4 days post-injury; (b) A front view of Anthony Stacey back at camp 4 days post-injury.

An X-ray the following day confirmed the doctor’s assessment of the injury. There was no further cause for concern for my well-being because there was no apparent bleeding inside the skull, and I was not having headaches. I rejoined the camp 4 days after the injury (see Figure 3). The camp doctor removed the sutures the following day while on a more leisurely and, for the doctor at least, more characteristic sailing activity.

### Frontal lobe damage

In 2008, a colleague began studying psychology at undergraduate level. During the first semester of lecturers, the curriculum included the anatomy of the brain and the functions of the various parts of the brain. Reflecting on their lived experience of me, they recognised a correspondence between the primary functions of the frontal lobe and some of my distinctive idiosyncratic behavioural and personality traits. The epiphany was that these traits may be related to my childhood head injury.

Computed tomography (CT) imaging was only invented very shortly after my head injury, and magnetic resonance imaging (MRI) was first performed on humans in 1977. Therefore, X-rays had been the only technology available to scan for possible brain trauma. Moreover, I had just entered adolescence when the injury occurred, and therefore I was at an early stage of the psychological and social transition between childhood and adulthood. Family and friends that observed behavioural or emotional changes over that period would have been attributed to adolescence. In retrospect, the injury could not have taken place at a worse time.

A suggestion of damage to my frontal lobe was evident even in my six-monthly high school reports. My schoolmasters reported with monotonous regularity that ‘*I had hoped for better results in his languages*’ or ‘*a definite improvement is required in his language subjects*’ and the like. Similarly, language comprehension has been extremely challenging, specifically in films and the lyrics in music. On the sports field, cognitive inflexibility (Vilkki 1992) was clearly evident in my inability to adapt to rapid changes in the state of play. Teachers noted that as a scholar, I had a good sense of what is ‘right and proper’ and that I stood up for my principles. This manifested in adulthood as being unreasonably rigid, inflexible and intolerant. There have been many instances in my life of my lack of sensitivity to situations or awareness of context, which has caused me and others embarrassment, both socially and professionally. I have experienced being emotionally unavailable through two unsuccessful marriages and parenting two children, and I am aware of often having good intentions but not getting things done. Situations in which extreme anger would have been an appropriate response only resulted in my being frustrated or indignant, due to an inability to experience anger. Some might label my preoccupation with recording and tracking my household water consumption, my household electricity consumption, my vehicles’ fuel consumption, my weight, my daily heartbeat, my exercise programme, etc. as obsessive. There is a well-established relationship between childhood traumatic brain injury and depression (see, for example, Albicini & McKinlay 2018; Anderson et al. 2011; Beauchamp et al. 2011; Mauri et al. 2014). This is consistent with my having experienced depression for all of my adult life, differing over time only in its severity.

### Botched surgery

Late in December 2008, I developed pain and inflammation in my upper right leg which a vascular surgeon diagnosed as thrombophlebitis. Their recommendation was that I undergo surgery to remove varicose veins, which then took place on 30 December. Within hours of surgery, the surgeon visited me in the ward; they told me that there had been ‘complications’ and that the surgical assistant had accidentally removed a section of my popliteal nerve. They explained that this would result in a ‘drop foot’ and that it would be a ‘significant’ permanent disability.

It is worth noting that notwithstanding the seriousness of my childhood head injury, I had never considered the notion that I was living with a disability. However, I was now living with an unequivocal physical disability. I notified the institution’s human resources division, who duly recorded me as ‘partially disabled’ on the human resources information system.

In the days and months that followed, I visited a variety of medical practitioners to identify what they could do to mitigate the disability. These included a neurosurgeon, orthopaedic surgeon, orthotist, biokineticist and podiatrist. The dysaesthetic pain was such that even the lightest touch – for example, the weight of a cotton bed sheet – was excruciating. The prescribed pain medication provided minimal relief and came with the loss of libido and depression as side effects. The loss of mobility interfered with intimacy and resulted in rapid weight gain and muscle atrophy. I struggled with everyday tasks such as getting dressed, bathing, walking barefoot, running, wearing sandals and even opening a pedal dustbin. By wearing an ankle–foot orthosis, I was able to learn to walk relatively normally. However, an asymmetric gait resulted in a sequestrated lumbar disc and an urgent laminectomy with some 3 months of recuperation. The long-term impact has been chronic lower back pain, intensified by remaining relatively inactive for extended periods, such as in ‘cocktail party’ settings, queuing, travelling and sleeping. In public, I had to become used to strangers staring at me when wearing shorts. Despite displaying the appropriate token, I regularly received verbal abuse and even physical threats for parking in bays reserved for persons with disabilities.

I felt neither anger nor malice towards the vascular surgeon, despite the profound impact I was experiencing on my day-to-day life. Nevertheless, I chose to seek compensation for medical expenses and loss of earnings. The medicolegal process involved consultations with a forensic actuary and examinations by a multiplicity of medical specialists. These included an orthopaedic surgeon, physiotherapist, clinical psychologist, biokineticist, occupational therapist, orthotist, neurosurgeons, vascular surgeon, neurologist and an industrial psychologist. After a gruelling and protracted process, the matter was finalised 4 years and 4 months after the injury.

### Performance of essential job functions

Social norms in the institution dictate that overt discrimination against persons with disabilities is unacceptable. Prejudice is nevertheless present even among those who mean well, described by Friedman (2018) as aversive ableism. My experience of parking bays on campus for persons with disabilities offers an obvious example. They are inadequate in number and many are in inappropriate locations. They are frequently used by persons without disabilities without sanction. Persons with nonobvious disabilities experience harassment and abuse for legitimate use, illustrating that prejudice is potentially greater against those with nonobvious disabilities.

In terms of the institution’s policies (University of the Witwatersrand 2016a, 2016b), an impairment must substantially limit one’s ability to perform the essential functions of one’s job in order to be deemed a person with disabilities. I have experienced the cruel irony that the more effective the rehabilitation, the less likely the institution is to accept one as a person with a disability. Notwithstanding the efficacy of rehabilitation, living with a disability reduces the amount of mental and physical energy available to perform in the workplace. This phenomenon has resulted in the popularisation of the neologism of spoon theory (Miserandino 2003) among persons with disabilities or chronic illnesses.

It became evident within weeks post-injury that I needed elevation and movement of my leg to tolerate air travel of more than 2 h. Travelling for more than 2 h in economy class resulted in the onset of severe nerve pain. Foreign travel for academic and research purposes, including conference attendance, is essential for an academic’s career advancement. Hence, unless I was appropriately accommodated, foreign travel would be a limitation on my ability to perform this essential function of my job.

### Ethical considerations

Ethical clearance was granted by the University of the Witwatersrand Human Research Ethics Committee (Non-Medical) H20/06/46.

## Results

### Personal injustices

In 2012, the relevant committee granted me 6 months sabbatical leave. I had two conference papers accepted for presentation at a European conference in June. I applied to the institution for funding to fly to the conference in business class to accommodate my need to elevate and have space to move my leg freely. After providing the necessary documentation and motivation, the powers that be agreed that the Faculty Research Committee budget would cover the costs of my travel. The vice chancellor of the institution then approved my application.

In 2013, I again had a conference paper accepted for presentation at a European conference. For the same reasons, I applied to the institution for similar funding. The relevant decision-maker initially approved my application but then retracted the decision on the grounds that I had cost the Faculty too much money the previous year. After extensive negotiations, approval for my travel was granted on the understanding that costs would be paid out of the School budget. I was subsequently advised that in the future, the additional cost of accommodation would be covered by the ‘reasonable accommodation’ clause in the institution’s disability policy.

I had no appetite for repeating the process for the following two years. I was exhausted and humiliated by the application and approval process, and I chose to spend four weeks in rehabilitation from depression and anxiety in 2014. In 2016, I applied for reasonable accommodation for foreign travel in terms of the institution’s policy, as I had been advised previously. The institution’s Disability Rights Unit convened a reasonable accommodation panel meeting, and the panel decided that, contrary to the previous directive, my application should rather be reviewed in line with the travel policy. This left the decision up to the faculty dean, who could approve business-class travel on the grounds of ‘a medical condition’ rather than disability. For the following two years, my foreign travel was approved in terms of the institution’s travel policy and paid from the School’s travel budget.

A new director of the School effectively deferred approval of travel to a European conference in 2018 to the faculty dean. Their position was that my own research funds had to cover the difference between economy and business class. I argued that would place an unfair additional burden on me to raise research funds, because the accommodation need was solely on account of my disability. The matter escalated first internally within the institution and then to the statutory dispute resolution body. The dispute remains unresolved to date but became somewhat moot as the coronavirus disease 2019 pandemic caused academia to adopt hybrid mode activities.

In 2019, two senior-level vacancies arose in the School. I considered myself a suitable candidate for either position. I therefore submitted applications through the appropriate channels and was shortlisted for both. During the first selection interview, the chairperson put to me that a referee had identified ‘administration’ as a weakness in my overall performance. I was completely blindsided by this revelation. Nobody had ever pointed this out to me in any performance review during some 18 years of employment with the institution. Weak administrative skills would be consistent with executive dysfunction. Realistically, the selection committee would not have taken into account my childhood head injury and symptoms of frontal lobe damage. The selection committee considered me to be ‘not appointable’ to the position.

Having made the connection between administrative skills and executive dysfunction, I reflected on the documented symptoms of frontal lobe damage. I wanted to understand the extent to which my lived experiences through adult life may be consistent with frontal lobe syndrome. I was able to positively identify many symptoms based on the definition by Krch (2011). I had previously been unaware of unintentionally creating false memories, disorganisation, inability to modify behaviour to accommodate new information, loss of spontaneity and risky behaviours. In social settings, I was unaware of diminished empathy, a limited range of emotions, aggression, irritability, social inappropriateness, tactlessness and poor self-awareness. These behaviours have evoked criticism, disapproval, anger, alienation and other forms of negative social feedback.

### Institutional policies related to disability

In this section, I cite examples from institutional policies that were applicable at the time, particularly those which are apparently well-meaning and beneficial but which constitute subtle or embedded discrimination against persons with disabilities.

The policies of the institution are administered within a policy framework and are subordinate to the *Higher Education Act* (Republic of South Africa 1997) and the statute of the institution. In terms of the policy framework, the registrar of the institution has been the executive responsible for disability rights. An objective of the disability policy has been to ensure disability integration into all human resource policies and procedures. It is therefore curious that the executive responsible for human resources and transformation has not been accountable for disability rights.

The policy framework also defines the role of the Institutional Forum, which is one of several structures to govern public universities for which the *Higher Education Act* provides:

Institutional (University) Forum advises council on implementation of the Act, race and gender policies, selection of senior management, codes of conduct, and fostering an appropriate institutional culture. (University of the Witwatersrand 2019:3)

Admittedly, the demographics of the institution suggest that there are fewer employees directly affected by disability policies than those who may be affected by race- and gender-related issues.^1^ However, it is problematic that the scope of such an important governance structure excludes disability policies. Similarly, various policies prescribe the composition of committees involved with the selection, appointment and promotion of employees. Generally, these policies refer to the representation only by race and gender, or they are silent on diversity and inclusivity requirements. One exception is the policy that states the following:

The selection committee must generally be representative in terms of race (35% black people), gender (50% female), persons with disabilities (where possible) and functional expertise. (University of the Witwatersrand 2016b:12)

That the committee should only be representative in terms of persons with disabilities ‘where possible’ implies that it may not be possible for a representative to represent persons with disabilities appropriately. This is a poor reflection on an institution with such a large number of employees. One of the explicitly stated principles of recruitment and selection is that:

Appointable candidates, who fulfil the definition of persons with disabilities in terms of the *Employment Equity Act*, shall be given preference regardless of whether they are internal or external. (University of the Witwatersrand 2016b:6)

On first impression, the effect of this clause is to implement an affirmative action measure designed to benefit candidates with disabilities. In practice, however, selection committees may not consider a candidate with disabilities to be ‘appointable’ because of the effect of their impairment, particularly in the case of nonobvious disabilities. In such cases, the above clause would not be deemed applicable. In other words, selection committees would need to assess candidates with disabilities to be ‘appointable’ *despite* their disabilities in order for those candidates to benefit from affirmative action measures. This places such individuals at an unfair disadvantage with respect to other candidates.

The ‘Reasonable Accommodation’ provisions of the disability policy begins by stating that the institution:

[…*R*]espects, and will implement the right of job applicants and employees with disabilities to receive reasonable accommodation, when the person voluntarily discloses a disability-related accommodation need. (University of the Witwatersrand 2016a:4)

Again, the obvious interpretation of this commitment is that all job applicants and employees with disabilities have a right to receive reasonable accommodation. However, the caveat is in the second clause, the implications of which are significant. Firstly, the person with disabilities may not necessarily be aware that their impairment is limiting their prospects. They will then be unaware of their eligibility to be considered for reasonable accommodation. Then, the institution accepts no responsibility for providing it, particularly in the case of nonobvious disabilities. This is especially unfair if the institution is aware of both the impairment and deficient performance but has failed to associate the two. Secondly, the person with disabilities may not necessarily be cognisant of reasonable accommodation measures that could potentially mitigate the effect of their impairment. Again, the institution accepts no responsibility for assisting the employee to identify such measures.

An alternative framing of the above commitment is that ‘The institution will not consider reasonable accommodation unless the person discloses a need related to their disability’. The upshot is that unless the employee themself articulates the associated need, disclosure of the disability itself initiates no affirmative action measures by the institution. The employee may thus be prejudiced as a direct consequence of their impairment.

### Disablist implementation of policies

In this section, I give examples of institutional policies that are well meaning, but which fail to achieve their intended beneficial outcomes because of a disablist institutional culture and environment.

Reverting to the recruitment, selection and appointment policy, and specifically the composition of the selection committee, the policy states that:

The Chair may co-opt additional members from within, or where this is not possible, external to the University to achieve race, gender and disability representivity. (University of the Witwatersrand 2016b:12)

This appears eminently reasonable to ensure diversity and inclusivity on the selection committee. The policy gives the chairperson the option of whether or not to achieve fair representation but creates no obligation on them to do so. The nature and diversity of disabilities are such that it is impossible for anyone to fully empathise with and appreciate the lived experience of those with disabilities. It is curious that the chairperson of a selection committee has the option not to include a person with disabilities, particularly if any one of the applicants lives with disabilities. Even if it were unintentional, constituents would consider the exclusion women or black people from the committee to be sexist or racist and unconscionable. Similarly, the exclusion or omission of persons with disabilities appears disablist and offensive.

The disability policy of the institution has included many ‘motherhood and apple pie’ statements – statements that encapsulate universally held values that no reasonable person would contest. By way of an example, the following is from the statement of commitment in the policy:

The [*Institution*] recognises that persons with disabilities have been historically marginalised in South Africa and that it is imperative that higher education, and [*the institution*] specifically, puts measures in place to redress the inequalities and disadvantages created by prejudice and discrimination, and contributes to a democratic vision which is respectful of all human rights. (University of the Witwatersrand 2016a:1)

**FIGURE 4:**
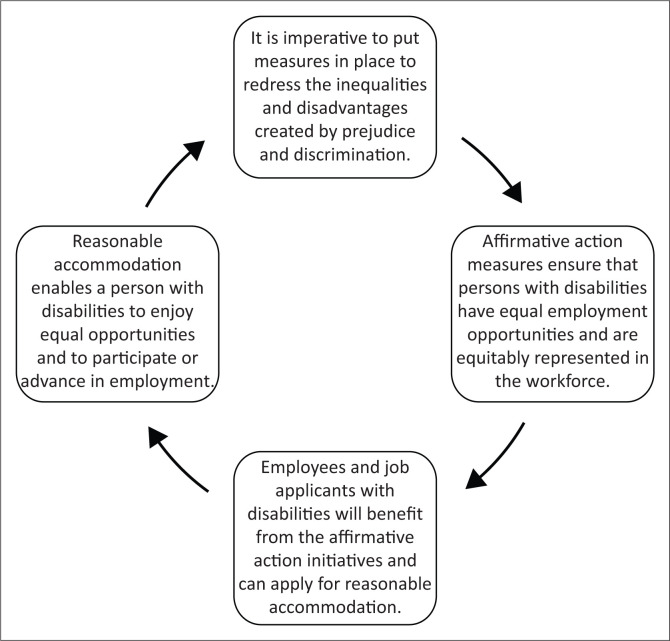
Circularity of affirmative action and reasonable accommodation.

Who could argue with that? However, in the words of a colleague who was a member of the selection committees, selection processes are ‘brutal’. Furthermore, Figure 4 illustrates that there is circularity in the following clauses of the policy:

‘Affirmative Action Measures’ are measures designed to ensure that suitably qualified persons from designated groups (i.e. persons with disabilities) have equal employment opportunities and are equitably represented in all occupational categories and levels in the [*institution’s*] workforce.

Employees and job applicants with disabilities will benefit from the affirmative action initiatives embarked upon by [*the institution*] and can apply for reasonable accommodation in accordance with the provisions of this policy. (University of the Witwatersrand 2016a:1)

And:

Reasonable accommodation is any change or modification made to an employment standard, policy, practice, job or the working environment which enables a person with a disability to enjoy equal opportunities with others and to have access to or to participate or advance in employment. (University of the Witwatersrand 2016a:15)

Ideally, this becomes a virtuous cycle from which persons with disabilities plus the institution itself benefit, but it requires adherence to both the letter and the spirit of the policy. Disablism disrupts the virtuous cycle, as the following examples illustrate.

The organisation’s disability policy has prescribed the composition and responsibilities of the Disability and Reasonable Accommodation Panel. It is significant that the policy has made no provision for discretion with regard to the inclusion of particular managers and an occupational health doctor. Specialists may be co-opted onto the panel should it be deemed necessary. The person disclosing their disabilities and applying for reasonable accommodation does not appear in front of the panel. This is problematic as it affords the individual no opportunity to verify that the panel is properly constituted. Even resorting to the *Promotion of Access to Information Act* (Republic of South Africa 2000) to access the minutes of the panel was unsuccessful, as the Act stipulates that:

The Information Officer may refuse a request for access to a record if the record contains … minutes of a meeting for the purpose of assisting to … take a decision in the exercise of a power or performance of a duty conferred or imposed by law … (s42 a)

It was impossible for me to substantiate that the panel that met in March 2016 to consider my application for reasonable accommodation excluded an appropriately qualified medical practitioner. The institution’s Disability Rights Unit may not have convened the panel in accordance with the institution’s disability policy. Persons *without* disabilities act as gatekeepers to reasonable accommodation for those with disabilities.

The precondition attached to reasonable accommodation is that it does not involve ‘unjustifiable hardship’. That is to say, an accommodation may not be unduly costly, expensive, substantial or disruptive, nor may it fundamentally alter the nature of the institution’s operations. An employer would have to take into account its financial circumstances and the estimated amount of expenditure required to accommodate when invoking the ‘unjustifiable expense’ defence. It is understandable that a small organisation may claim unjustifiable hardship for even a modest accommodation need. However, that defence from an organisation with an expenditure of more than R 4.5 billion (approximately USD 260 million) is implausible. The institution’s disability policy has stated that:

A central budget through the ‘Reasonable Accommodation Fund’ administered by the Disability Rights Unit will be made available to meet the accommodation requirements of the [institution]. (University of the Witwatersrand 2016a:4)

And:

All Faculties and Divisions will be required to progressively integrate reasonable accommodation in their budgeting processes. (University of the Witwatersrand 2016a:4)

Unbudgeted expenditure is anathema to the institution. The institution can therefore invoke the ‘unjustifiable hardship’ defence in any reasonable accommodation case in two ways: firstly, by overlooking the policy that provides for a central budget; secondly, by accepting Faculties’ and Divisions’ noncompliance with the requirement to integrate reasonable accommodation into their budgeting processes.

The institution’s disability policy explicitly acknowledged that confidentiality is an important ‘guiding principle’ which should be respected at all times. However, confidentiality remains only a principle because it is wholly impracticable. By way of example:

- Line managers, Human Resources and the Transformation Office are all represented on the reasonable accommodation panel.
- Line managers have to be apprised of person’s disability status if they are to budget for reasonable accommodation.
- Finance personnel have to administer any expenditure on reasonable accommodation.
- Selection and promotion committees, including applicants’ peers, have to be apprised of an applicant’s disability status to take this into account in their decision-making.
- Employee relations and senior executives are involved in the event of disputes arising from interpretation of the policy.

The plethora of colleagues who have knowledge of my disabilities has made a mockery of the confidentiality principle.

Self-representation by persons with disabilities is widely considered to be an essential element of disability rights (Waldschmidt et al. 2015), as captured in the slogan ‘nothing about us without us’. Indeed, the disability policy incorporated the principle of self-representation in the clause stating that:

The process of identifying and implementing reasonable accommodation should involve consultation with different role players including the person seeking the accommodation in an effort to reach a mutually acceptable solution. (University of the Witwatersrand 2016a:17)

It is therefore inexcusable for a reasonable accommodation panel to take and implement decisions without consulting the person with disabilities, as happened with each of my reasonable accommodation applications. Similarly, it is improper and insensitive to appoint a person without disabilities to represent those with disability in any governance structure of the institution.

The inappropriateness, obsolescence and negativity associated with the widely discredited medicalisation of disability are widely acknowledged (e.g. Department of Social Development 2016; Hayes & Hannold 2007; Hogan 2019; Shakespeare 2006). Yet in response to my request for travel-related reasonable accommodation, the outcome of the panel meeting was that:

[… *A*]ll applications for travel related reasonable accommodation should be reviewed in line with [*the institution’s*] travel policy and that a member of [*the executive*] can make such a decision in line with [*the institution’s*] travel policy. (University of the Witwatersrand [*Higher Education Institution*] Disability Reasonable Accommodation panel, pers. commun., 22 August 2019)

The relevant paragraph of the institution’s travel policy read as follows:

The Vice Chancellor and / or [*senior executive*] may or may not approve premium economy or business class for other staff members under the following circumstances: … Extenuating circumstances exist, such as a severe medical condition (supported by a medical certificate). (University of the Witwatersrand 2020:5)

Policies have been silent on what constitutes a ‘severe medical condition’ as opposed to a disability. In my application, the decision of whether or not to provide reasonable accommodation was deferred to an executive who is unlikely to have any expert knowledge of my particular disability. The subjective judgement of severity and disability is the prerogative of an able-bodied executive. Expenditure related to providing accommodation in the absence of a central budget would have to come out of the senior executive’s faculty funds and would be unbudgeted. This would be a significant disincentive for them to approve the requested reasonable accommodation. This is little different from the cuts in social benefits being experienced by those with anything but the most severe impairments (Oliver 2013) and, by extension, those with nonobvious disabilities.

## Discussion

### Psychosocial consequences

I have discerned three distinct elements of my responses to my experiences. Firstly, I express judgements about those individuals involved with the implementation of disability policies in the institution. Secondly, I formulate judgements of their actions and behaviour, and finally, I articulate experiences that are an integral part of my life with disabilities.

My judgements of the executives and managers tasked with implementing disability policies are that they are not malicious or spiteful individuals. I think that they perceive the priorities and demands on the institution to be more pressing. They appear to lack empathy, compassion and a willingness to develop an awareness of the lived experiences of those with disabilities. As a collective, they demonstrate aversive disablism (Deal 2007), as hurtful as aversive racism (Dovidio, Gaertner & Pearson 2017) and aversive sexism (Tracy & Rivera 2010). Perhaps it is not surprising that feelings tend to be intellectualised in an institution led by academics and researchers – a tendency referred to as *post-emotionalism* (Rodger 2003).

The actions and behaviours of those individuals had a profound impact on me. My judgement is that the consequences were unintended but predictable due to the disablist institutional culture. The lack of adequate policy and pragmatic measures to protect my confidentiality mentioned earlier was deeply disrespectful. The reframing of my physical impairment as merely a medical condition that does not qualify for reasonable accommodation is a form of manipulation. It gives an unqualified person the power and authority to act as a gatekeeper. I am gaslighted (Abramson 2014) by the implication that my experience of living with a specific disability is mistaken and imaginary. Each and every microaggression has wounded, humiliated and retraumatised me.

The policies of the institution give substance to the psychological contract (Rousseau 1989) between employer and employee. Affirmative action entitles members of designated groups to preferential treatment over others, and employees with disabilities therefore have a reasonable and greater sense of entitlement. Priesemuth and Taylor (2016) found a positive relationship between the strength of entitlement and the severity of depressive moods when expectations are unmet or promises broken. It follows, therefore, that employees with disabilities are at greater risk when the psychological contract is broken through the implementation of a policy that is perceived to be unfair.

Expenditure by the institution on reasonable accommodation has been prioritised on the basis of strict managerialist criteria such as ‘… inter alia, costs, budgetary resources, opportunity costs, [and] relevance’. The moral imperative to create equal opportunities for employees with disabilities is absent. Executives and managers have misconstrued their managerial discretion by prioritising expenditure ahead of reasonable accommodation. They have been remiss in not adhering to the policy to make available a central budget administered by the Disability Rights Unit. In doing so, they have sanitised and protected the community from the discomfort of engaging with disability. Discrimination against persons with disabilities has been perpetuated, and the culture of disablism pervading the institution has been reinforced.

The dominant response that I have experienced as a result of analysing the events, policies and responses through the course of this study has been a sense of loss. Until a few years ago, I had a somewhat postapocalyptic view of the world and society. My experience of living was that it was inescapably hostile, unsafe, confusing, competitive, dangerous, irrational, brutal, random, deceitful, dark and toxic. The sense of loss comes from the recent realisation that I have viewed life through an imperfect lens for so long. Sadly, this has had a detrimental influence on my life and on those closest to me.

I acknowledge that I allowed myself to become excessively invested in the institution which has left me exposed, vulnerable and disillusioned. I think that I have been singled out because of activism and leadership within organised labour. I trusted in the morality, integrity and decency of the institution as manifest in its policies and procedures, but instead of being affirmed and accommodated, my trust has been betrayed. I am frustrated that disablism prevails, and as a David I am powerless against the Goliath of the institution.

## Conclusion

The study has drawn attention to some challenging findings. The management of the institution may want to consider why, with such a large number of employees, it acknowledges that it may not be possible for persons with disabilities to be appropriately represented on selection committees. Similarly, it should consider why candidates with disabilities should be disadvantaged by having to demonstrate themselves appointable despite their disabilities. Then, there is surely an obligation on managers who are aware of substandard performance by an employee with disabilities to advise the individual of their right to apply for accommodations.

The purpose of this study is not to attract sympathy, pity or validation. I also have no desire to carry out an *ad institutum* attack or ‘culture assassination’ of the institution. My intention is rather to draw attention to challenges that are experienced by both employers and their employees with disabilities. Greater awareness can lead to mutually beneficial improvements in institutional policies and the relationships between employers and their employees with disabilities.

It appears as if the implementation of disability policies is guided by managerialism (Shepherd 2018) and that the letter of the policies takes precedence over the spirit and intent. Experience is that policy implementation is not necessarily successful in promoting access to full participation in all aspects of the institution, because not all discriminatory barriers are effectively removed. Persons with disabilities do not achieve self-representation because policies are silent on the matter or because such provisions that are in policies go unheeded. The institution is relinquishing the benefits of a diverse workforce that values the unique insights, experiences and knowledge of persons with disabilities.

I have described the unintended impact of the manner in which management has implemented policies as generally detrimental to the emotional well-being of employees with disabilities. This is a result of the perception that management has broken the implicit promise embedded in the institution’s policies. Violations of the psychological contract have been found to be positively correlated with employee turnover and negatively with trust, satisfaction and intentions to remain (Robinson & Rousseau 1994). All these consequences are likely to be detrimental to the institution. Maybe more pragmatic (less progressive) policies are preferable to mitigate against unfulfilled expectations of affirmative action and accommodation.

**FIGURE 5:**
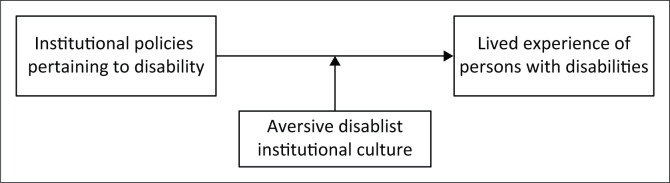
Model of the moderating effect of disablism on the relationship between policies and lived experience.

Aversive disablism neutralises the intended and potential benefits of institutional policies on the lived experience, well-being and quality of life of employees with disabilities. Figure 5 illustrates a model of the moderating effect of an aversive disablist institutional culture on the relationship between policies and the lived experience of employees with disability. This alludes to the opportunity and need for future research into this phenomenon.

In closing, the ‘black dog’ of depression (Howard 1992) is my lifelong companion. However, I am no longer afraid of its ferocity because we have found a way of living together in harmony.

## Epilogue

The institution has recently amended its policy affecting the inclusion of persons with disabilities in the workplace. The revised policy still regards disability as anomalous and requiring treatment rather than being a normal phenomenon within a community. This is evidence that ableism is self-perpetuating. While disablism may only be evident in the operationalisation of policies, ableism would appear to be embedded in policy frameworks. This has important implications for institutions and warrants further research.

## Footnotes

1 Less than 1% of the institution’s employees have disclosed disabilities, compared to the South African national prevalence of 7.5% (Statistics South Africa 2014).
